# Bouveret’s Syndrome: A Case Series and Literature Review on a Gallstone Disease Causing Gastric Outlet Obstruction

**DOI:** 10.7759/cureus.27519

**Published:** 2022-07-31

**Authors:** Adlene I Adnan, Osborne P Vaz, Snehal Lapsia, Asma Sultana, Mooyad A Ahmed

**Affiliations:** 1 Medicine, Leeds Teaching Hospitals NHS Trust, Leeds, GBR; 2 General Surgery, Lancashire Teaching Hospitals NHS Foundation Trust, Preston, GBR; 3 Radiology, East Lancashire Hospitals NHS Trust, Blackburn, GBR; 4 General Surgery, East Lancashire Hospitals NHS Trust, Blackburn, GBR; 5 Colorectal Surgery, Royal Blackburn Hospital, Blackburn, GBR

**Keywords:** bouveret’s syndrome, gallstone ileus, obstruction, endoscopy, surgery, endoscopic retrograde cholangiopancreatography (ercp), laparoscopic cholecystectomy. clip migration. hem-o-lok clip. common bile duct (cbd). endoscopic retrograde cholangiopancreatography (ercp). magnetic resonance cholangiopancreatography (mrcp)

## Abstract

Introduction

Bouveret’s syndrome refers to a gastric outlet obstruction due to the impaction of a large gallstone following retrograde migration via a bilio-duodenal fistula. Although no clear management guideline has been formulated, different treatment modalities have been described, including endoscopic stone removal using classical endoscopic devices, like snares and forceps, or fragmentation of stones with new devices, such as lasers and extracorporeal shockwave lithotripsy (ESWL).

Results

This case series reports six patients who have been diagnosed with Bouveret’s syndrome and have presented with interesting radiological and endoscopic findings. The report is followed by a literature review, including diagnostic and management options for this rare condition.

Discussion

Cholelithiasis is a common condition occurring in the general population and may develop rare complications such as cholecystoduodenal fistula. Bouveret’s syndrome presents with a clinical picture similar to that of gastric outlet obstruction, and laboratory findings are often consistent with an obstructive jaundice picture. The use of endoscopic treatment with a range of different lithotripsy modalities has been described to manage this condition.

Conclusion

The diagnosis of Bouveret’s syndrome is made after performing appropriate imaging studies. The first-line management option is endoscopic treatment. If this fails, surgical intervention is recommended.

## Introduction

Bouveret’s syndrome is a rare condition of gastric outlet obstruction due to the impaction of a large gallstone in the pylorus following retrograde migration from the duodenum. The gallstone erodes through the gallbladder wall into the adjacent structures such as the duodenum and stomach, forming a cholecystoenteric fistula. These include cholecystoduodenal (most common), cholecystogastric, cholecystocolic, and choledochoduodenal fistula [1,2]. Cholecystodoudenal fistula was first reported by Beaussier in 1770 and in 1896, and then it was the French physician, Leon Bouveret, who officially published two reports on patients who had gastric outlet obstruction secondary to gallstone impaction in the duodenum. Following this, there have been a few case reports of distinctive manifestations of Bouveret Syndrome as well as its various endoscopic treatment modalities [2,3].

The incidence of Bouveret’s syndrome is higher in women (65%) [3]. Patients with Bouveret's syndrome are commonly elderly (median age of 74.1 years old), and hence have a higher chance of presenting to the hospital with other comorbidities related to old age. This increases the likelihood of patients presenting with this condition being frail and having multiple comorbidities. This links the condition with an increased risk of mortality [3]. Surgical intervention is associated with increased mortality by 7.7%, which suggests less invasive interventions such as endoscopic intervention and stone removal should be attempted first [4]. If endoscopic extraction has failed, the options that are available are simple enterolithotomy, duodenotomy or gastrotomy, and stone extraction [3].

We have compiled several cases of Bouveret’s syndrome with remarkable radiological findings that have been successfully managed through either endoscopic treatment or surgical intervention. This is followed by a systematic review of the literature.

This article was previously presented as a meeting abstract at the international conference called Associations of Surgeons in Training (ASIT) from March 5th to March 7th, 2021, and the abstract accepted for a poster presentation was published in the British Journal of Surgery (BJD).

## Materials and methods

A thorough literature search was performed on PubMed, Medline (Ovid), Academic Research, Google Scholar, and other medical journals on Bouveret's Syndrome and its management plan. It was noted that there is no clear treatment guideline for this condition, but different interventions have been described.

In this research, several interesting cases of Bouveret's syndrome with remarkable radiological findings have been compiled and discussed. This case series demonstrated six patients with this condition, who have been successfully treated through a variety of treatment modalities depending on their clinical condition, health background, and imaging findings. This is followed by a review of the literature to compare the management plans of other known Bouveret's syndrome cases with the six cases that have been observed in clinical practice to formulate a clear management plan for this condition.

## Results

Case 1

A 74-year-old Caucasian male was admitted with a two-week history of right upper quadrant pain, dark urine, and a two-day history of haemetemesis. He had no other associated symptoms. His past medical history included calculous cholecystitis, for which he was awaiting a laparoscopic cholecystectomy, Parkinson’s Disease, atrial fibrillation, and transurethral resection for benign prostate hyperplasia. On physical examination, the patient had a soft, but distended abdomen. He was tender in the right hypochondrium and epigastrium.

On admission, the patient's laboratory results were seen (Table 1). He was appropriately resuscitated with intravenous (IV) fluids; he was started on IV antibiotics; and his clopidogrel was omitted. He received a blood transfusion to treat his anaemia. He had an ultrasound scan of the abdomen which showed calculus cholecystitis with a dilated common bile duct of 7.4 mm. He behaved clinically as having cholangitis.

**Table 1:** On admission laboratory blood results of the patient in case 1. g/L: gram per litre; 10^9^/L: 10^9^ per litre; µmol/L: micromole per litre; IU/L: international unit per litre; mg/L: milligram per litre. Hb: haemoglobin, WBC: white blood cell count, ALT: alkaline transaminase, AST: alkaline phosphatase, CRP: C-reactive protein.

Component	Results	Normal reference range and units
Hb	79	115–165 g/L
WBC count	14.7	4.0–11.0 × 10^9^/L
Neutrophils	12.2	2.0–7.5 × 10^9^/L
Bilirubin	23	0–21 µmol/L
ALT	120	0–34 IU/L
ALP	341	30–130 IU/L
CRP	136	0–10 mg/L

**Figure 1:**
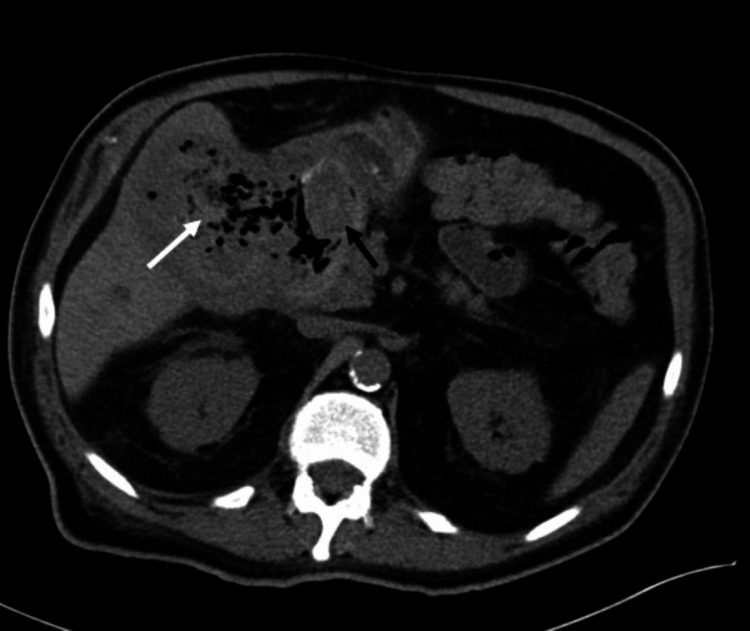
Axial CT non-contrast demonstrates gallstone (black arrow) fistulated into duodenum with gas in the gallbladder (white arrow).

**Figure 2:**
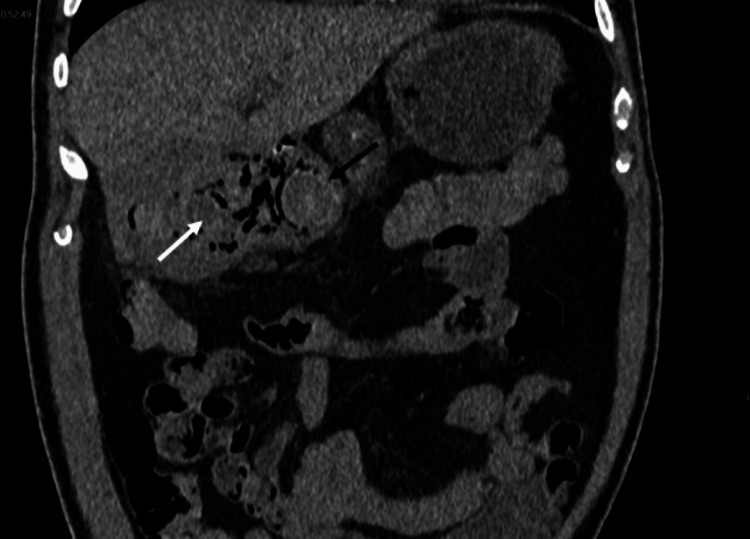
Coronal CT non-contrast, as previous image, demonstrates gallstone (black arrow) fistulated into duodenum with gas in the gall bladder (white arrow).

On day 3 of admission, he had further haematemesis (approximately 500 ml). He was transfused an additional unit of blood, started on tranexamic acid, and an inpatient oesophago-gastroduodenoscopy (OGD) was performed, which revealed that his stomach contained a large amount of blood and biliary stone material with clots occupying the pylorus and first section of the duodenum (D1). He was injected with 14 mililitre (mL) of adrenaline 1/10,000, then a hemostatic powder agent, haemospray, was used to achieve haemostasis. Haemospray is used endoscopically to produce an adhesive barrier with the bleeding site, and therefore, it forms a mechanical barrier to stop further bleeding. CT angiography was performed on the same day; it showed cholecystodoudenal fistula with stone eroding into D1 (Figures 1-2). The patient underwent endoscopic retrieval of the calculous and a naso-jejunal tube was placed for feeding. Subsequently, the patient made a steady recovery and was safely discharged home.

Case 2

An 88-year-old Caucasian male was admitted with one week of abdominal pain and a three-day history of coffee ground vomit. He reported poor oral intake for several days prior to admission. On physical examination, he displayed mild tenderness in the epigastric region, but the entire abdomen was soft. He had several comorbidities which included chronic kidney disease, anterior resection for large bowel cancer seven years ago, glaucoma, a coronary artery bypass graft, and aortic valve replacement for severe aortic stenosis eight years prior to presentation.

**Figure 3:**
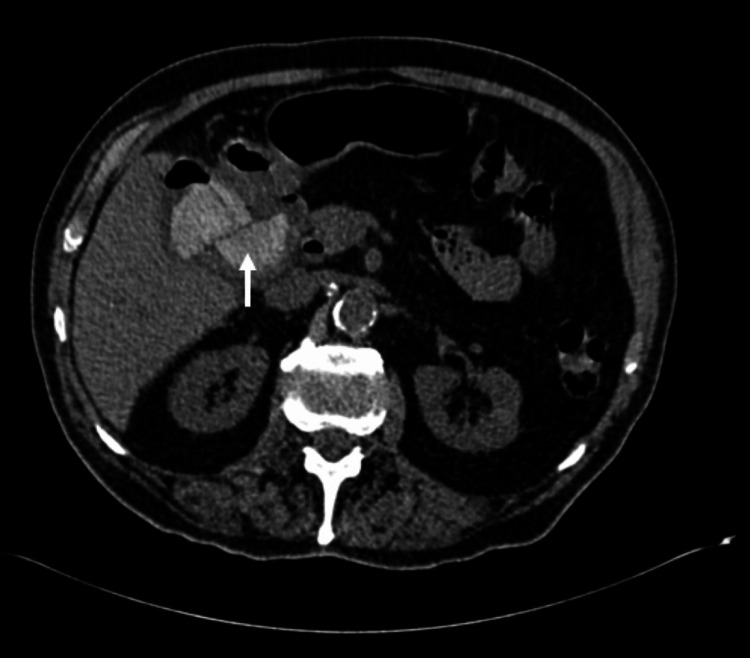
Axial CT non-contrast gallstone within the lumen of the duodenum (white arrow).

**Figure 4:**
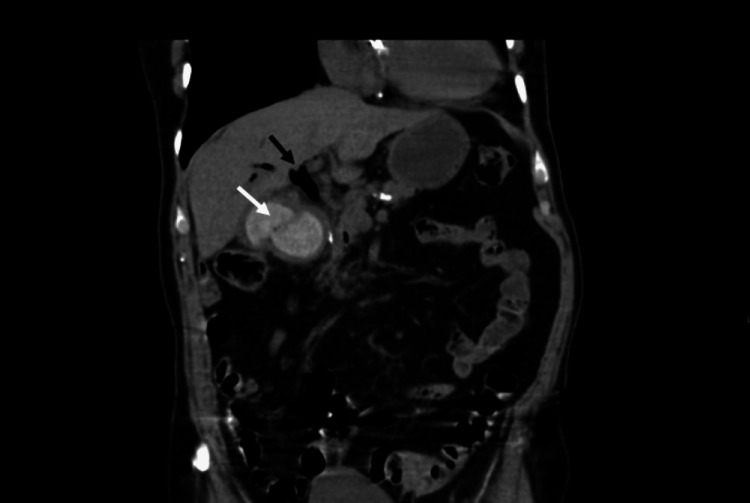
Coronal CT showing gallstone within the lumen of the duodenum (white arrow) and also note gas within the common bile duct (black arrow).

On admission, the patient's blood results were seen (Table 2). He was treated with intravenous fluids, antiemetics, and analgesia. On day 3 of admission, his OGD showed a large amount of black fluid filling the stomach, severe oesophagitis, and large non-bleeding ulceration with a large brown foreign body in D1, causing gastric outlet obstruction. The foreign body could not be extracted during the OGD. Subsequently, the patient had a CT abdomen and pelvis with contrast, which revealed that he had a gallstone eroding through his gallbladder into D1/2 (Figures 3-4). On day 4, his Hb had dropped to 80 g/l from 110 g/l the previous day, and he was immediately resuscitated with a blood transfusion. He had a repeat OGD where the foreign body was identified as a gallstone. Stone extraction was attempted; however, it was not successful even with the use of an 18-mm balloon and snare. A duodenal stent was subsequently inserted to bypass the obstruction due to the impacted gallstone and to bypass the obstruction. Patency was confirmed by a contrast study, and then the patient gradually recovered and was discharged home.

**Table 2:** On admission laboratory blood results of patient in case 2. g/L: gram per litre; 10^9^/L: 10^9^ per litre; µmol/L: micromole per litre; IU/L: international unit per litre; mg/L: milligram per litre. Hb: haemoglobin, WBC: white blood cell count, ALT: alkaline transaminase, AST: alkaline phosphatase, CRP: C-reactive protein.

Component	Results	Normal reference range and units
Hb	110	115–165 g/L
WBC	15.1	4.0–11.0 x 10^9^/L
Neutrophils	12.2	2.0–7.5 × 10^9^/L
Bilirubin	20	0–21 µmol/L
ALT	32	0–34 IU/L
ALP	97	30–130 IU/L
CRP	35	0–10 mg/L

A year later, he was admitted for intermittent epigastric pain; this was initially thought to be due to displacement of the duodenal stent, but it was ruled out on an abdominal X-ray. He continued to have three further admissions to the hospital, once for cholangitis and twice for biliary sepsis. The latter was treated with antibiotics, and in both admissions for biliary sepsis, he was discussed at the upper Gastro-Intestinal Multidisciplinary Meeting (MDT). However, in both instances, it was deemed too high-risk for any intervention to be performed.

Case 3

An 81-year-old Caucasian female was admitted with a one-week history of bilious vomiting. She had no history of abdominal pain or fever. She opened her bowels four days prior to admission. On examination, her abdomen was soft, with no signs of peritonism. Rectal examination revealed an empty rectum with no masses felt. Her background medical history consisted of gallstones, for which she was awaiting a laparoscopic cholecystectomy, iron deficiency anaemia, duodenal polyps, lymphoedema and hypothyroidism. She had no previous surgical history.

Upon admission, her laboratory results were seen (Table 3) as having an acute kidney injury (AKI)-Stage 3. She was immediately resuscitated with intravenous fluids for her AKI, her nephrotoxic drugs were withheld, and she was catheterised to monitor urine output. A chest X-ray was performed on admission and was unremarkable. An abdominal X-ray, however, showed dilated small bowel loops, in keeping with paralytic ileus, which was treated conservatively with the insertion of a nasogastric (NG) tube and administration of intravenous fluids. Subsequently, the patient improved clinically.

**Table 3:** On admission laboratory blood results of patient in case 3. g/L: gram per litre, 10^9^/L: 10^9^ per litre, µmol/L: micromole per litre, IU/L: international unit per litre, mg/L: milligram per litre, mmol/L: millimole per litre, ml/min/1.73 m^2^: millilitre per minute per 1.73 metre square. Hb: haemoglobin, WBC: white blood cell count, ALT: alkaline transaminase, AST: alkaline phosphatase, CRP: C-reactive protein, eGFR: estimated glomerular filtration rate.

Component	Results	Normal reference range and units
Hb	188	115–165 g/L
WBC count	15.1	4.0–11.0 × 10^9^/L
Neutrophils	12.2	2.0–7.5 × 10^9^/L
Bilirubin	22	0–21 µmol/L
ALT	57	0–34 IU/L
ALP	260	30–130 IU/L
CRP	19	0–10 mg/L
Sodium (Na)	135	133–146 mmol/L
Potassium (K)	4.5	3.5–5.3 mmol/L
Urea	37	2.5–7.8 mmol/L
Creatinine	300	46–92 µmol/L
eGFR	12	More than 90 ml/min/1.73 m^2^

**Figure 5:**
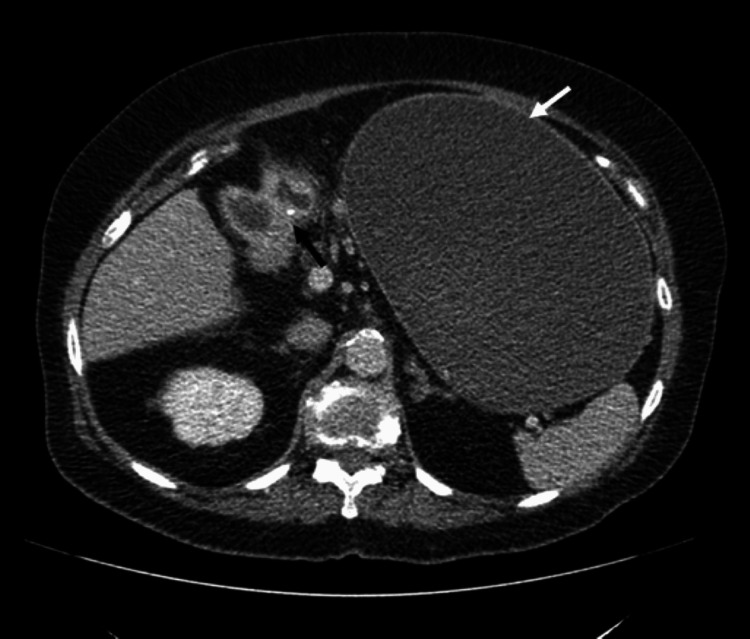
Axial portal venous CT shows gallbladder adherent to duodenum with a calcified opacity representing a gallstone within the duodenal lumen (black arrow) and massive gastric distension (white arrow).

**Figure 6:**
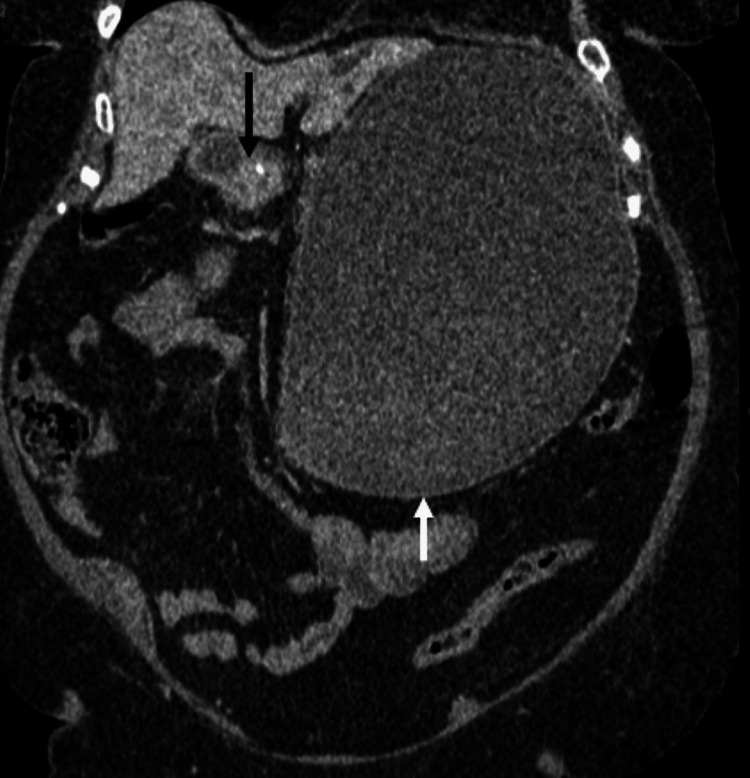
Coronal portal venous CT, as previous image, showing gallbladder adherent to duodenum with a calcified opacity representing a gallstone within the duodenal lumen (black arrow). Also note massive gastric distension (white arrow).

**Figure 7:**
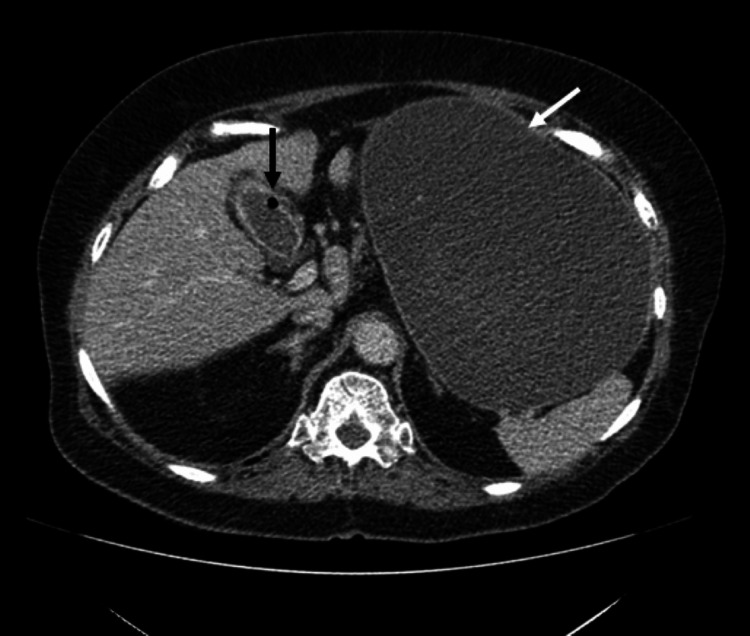
Axial portal venous CT shows speck of gas in the gallbladder in keeping with a fistula (black arrow). Massive gastric distension again noted (white arrow).

On day 5 of admission, her AKI had improved significantly; with laboratory results showing urea of 4.2 mmol/L, creatinine of 53 µmol/L, and eGFR of 86 mL/min/1.73 m^2^. A CT thorax, abdomen, and pelvis (CT TAP) was performed and it revealed a gallstone ileus with distal small bowel obstruction. Her gallbladder containing air had collapsed with a large fistula communicating with the duodenal cap. There were two non-obstructing stones within the second and third part of the duodenum; two gallstones in a loop of small bowel within the pelvis; and finally, a large laminated stone measuring approximately 3.7 cm, which was causing bowel obstruction with small bowel loops fluid-filled and dilated proximal to it (Figures 5-7). On the same day, she was operated on and had an exploratory laparotomy, an enterotomy, and the extraction of three gallstones. The gall bladder was left without intervention.

Post-operatively, the patient had myocardial infarction; the electrocardiogram (ECG) showed small T waves at V1 to V3 and ST depression on leads I, II, and AVL. Troponin was raised to 2124 ng/L. However, the patient remained asymptomatic. She was reviewed by the cardiology team. She was then treated conservatively. Her troponin levels were decreasing steadily. The patient made a good recovery and was discharged home on day 3 post-operation.

Case 4

A 79-year-old Caucasian female was transferred from a different trust hospital. She was initially presented with a one-week history of swallowing difficulties. She presented with poor appetite and lethargy and had lost 6.35 kg within three weeks. She felt nauseous and had episodes of vomiting and bringing out brown vomitus. Her bowels had not been opened for one week and she did not pass any flatus. Her past medical history included type 2 diabetes mellitus and hypertension. Her initial blood results showed that her liver function test and inflammatory markers were within the normal range.

**Figure 8:**
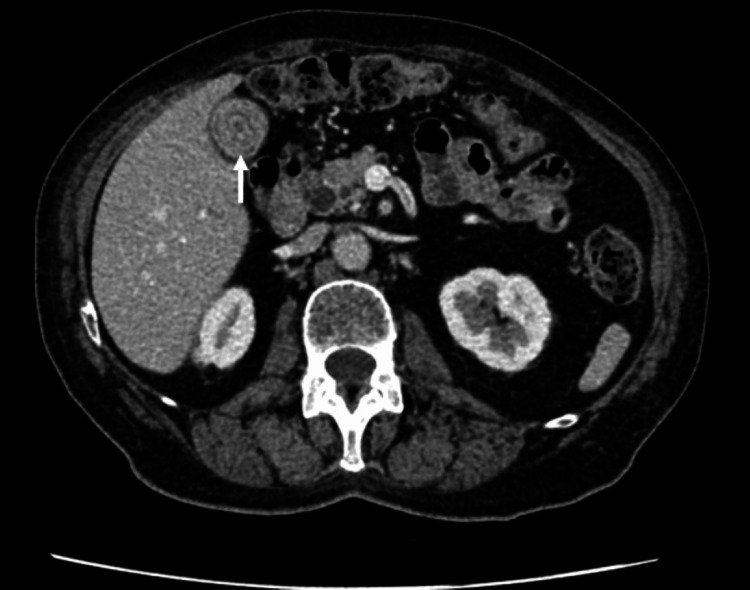
Axial portal venous CT shows a gallstone within the gallbladder (white arrow) with no acute changes.

**Figure 9:**
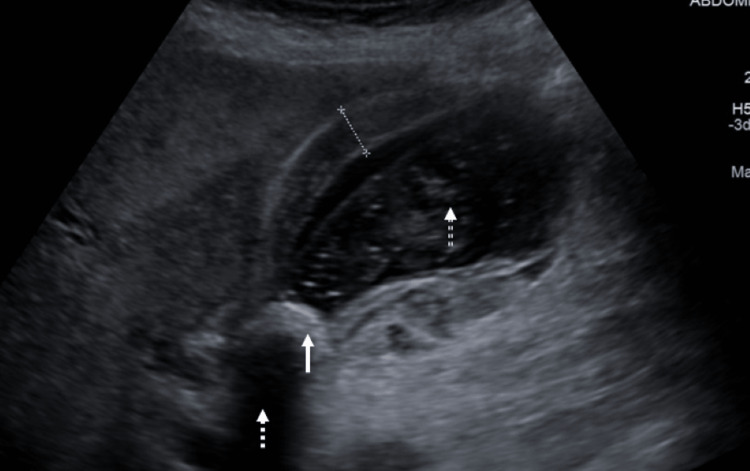
Ultrasound scan at the time symptoms demonstrates gallstone (white arrow) in duodenal lumen with posterior acoustic shadowing (white dashed arrow) and proximal dilation (white double line dashed arrow).

A CT thorax, abdomen, and pelvis were done two days prior to admission as an outpatient CT scan, and it showed the presence of a gallstone with the gallbladder and there were no acute changes (Figure 8). Prior to her hospital transfer, an OGD was performed for her new onset of dysphagia and weight loss. It revealed mild grade oesophagitis and a benign-looking stricture in the first part of the duodenum, likely to be from external compression. An ultrasound scan of the abdomen demonstrated a gallstone in the duodenal lumen with proximal dilation (Figure 9). The gallstone had impacted the duodenum, causing gastric outlet obstruction. Therefore, it was decided for her to have a subtotal cholecystectomy procedure.

As such, she was transferred to have the procedure done. Upon admission, she was stable. Her abdominal examination was soft, non-tender, and showed no signs of peritonism. Her bowel sounds were also present. She was initially managed with the insertion of an NG tube, given intravenous fluids, venous thromboembolism prophylaxis, and total parenteral nutrition.

On day 2 of admission, she underwent a laparoscopic subtotal cholecystectomy for choleduodenal fistula. Her gallbladder was opened surgically and two large obstructing stones were removed. On removal of the stones, a fistula connecting to the duodenum was visualised with the opening of the cystic duct. Then, a subtotal cholecystectomy was done. She had a drain in situ.

On day 10 post-operation, the patient had not opened her bowels since she had the surgery and had one episode of clear vomiting. She had an oral contrast medium, Gastrogaffin, with abdominal X-ray scans that showed no mechanical obstruction, indicating that the patient had developed post-operative ileus. She eventually recovered and improved clinically to be discharged home. The patient had a prolonged hospital stay due to prolonged ileus. The patient eventually recovered and was discharged home well.

Case 5

A 53-year-old South Asian male was admitted due to one day of sudden onset vomiting and central abdominal pain that radiated to the back and two days of constipation, but was passing flatus. He had been feeling generally unwell for two weeks, with unintentional weight loss over four weeks. On examination, there was a midline scar from a previous right hemicolectomy for colon cancer. His abdomen was slightly distended and mildly tender all over, otherwise soft with the presence of a known paraumbilical hernia. Bowel sounds were reduced and a rectal examination revealed a small amount of faeces in the rectum. His medical background included previous spinal surgery in 2007, chronic back pain, Bell’s palsy, bowel cancer hemicolectomy, and adjuvant chemotherapy.

**Figure 10:**
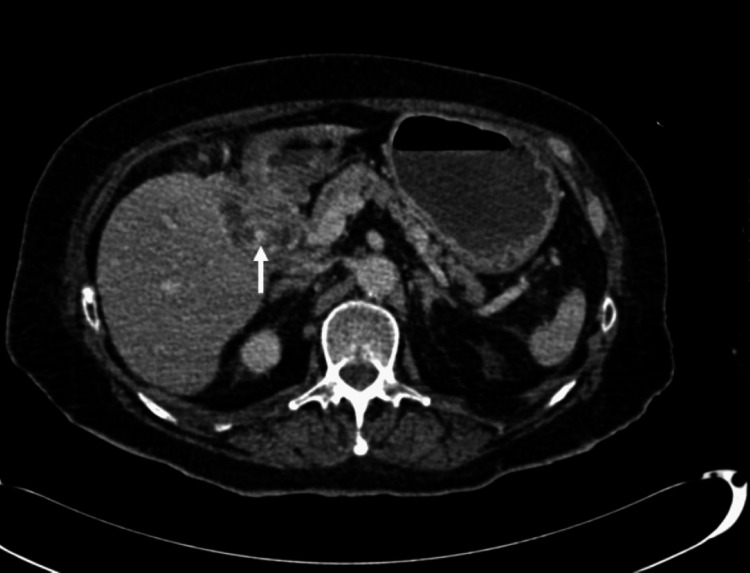
Axial portal venous CT shows gallstone fistulated into the duodenum (white arrow) with gastric dilatation.

On admission, his blood results were seen (Table 4). He was resuscitated with intravenous fluids and started on analgesia and antibiotics. He had a CT abdomen and pelvis with intravenous contrast which revealed a complex appearance with a possible fistulous connection between the gallbladder and the second part of the duodenum with a centrally calcified stone within the duodenum (Figure 10).

**Table 4:** Laboratory blood results of patient in case 5. g/L: gram per litre; 10^9^/L: 10^9^ per litre; µmol/L: micromole per litre; IU/L: international unit per litre; mg/L: milligram per litre; mmol/L: millimole per litre. Hb: haemoglobin, WBC: white blood cell count, ALT: alkaline transaminase, AST: alkaline phosphatase, CRP: C-reactive protein.

Component	Results	Normal Reference Range and Units
Hb	145	115–165 g/L
WBC count	12.9	4.0–11.0 x 10^9^/L
Neutrophils	9.9	2.0–7.5 x 10^9^/L
Bilirubin	17	0– 1 µmol/L
ALT	47	0–34 IU/L
ALP	187	30–130 IU/L
CRP	135	0–10 mg/L
Sodium (Na)	144	133–146 mmol/L
Potassium (K)	4.6	3.5–5.3 mmol/L
Urea	7.0	2.5–7.8 mmol/L
Creatinine	72	46–92 µmol/L

The patient was treated conservatively with nasogastric drainage and intravenous fluids followed by an OGD on day two. In the duodenal bulb, there was a fistula in keeping with the cholecystoduodenal fistula. There was a large (more than 3 cm) stone that was impacted in the duodenal bulb to either come out of the pylorus or go to D2, as the stone had narrowed the lumen of D2. The endoscopist tried to retrieve the stone with foreign body forceps, a Roth net, and a trapezoid basket. However, it was not successful due to its position and mobility. Eventually, the endoscopist managed to push the large stone gently into D2. The stone then could not be visualised and it may have possibly been pushed through into the small bowel.

Post-operatively, the patient was clinically improving and he continued on conservative management. On day 5 of admission, his blood results improved with a CRP of 37 mg/L, liver profile (bilirubin of 8 µmol/L, ALT 36 IU/L, ALP 125 IU/L, albumin 35 g/L), and urea and electrolyte tests were within normal ranges. His Hb was 126 g/L, WBC was 7.4 × 10^9^/L, and neutrophils were 5.0 × 10^9^/L. Subsequently, the patient was optimised and medically fit for discharge on day 8 of admission.

Case 6

A 73-year-old Caucasian female was admitted with generalised abdominal pain, diarrhoea, and a reduced appetite for five days. She had experienced similar abdominal pain three weeks ago. On examination, her abdomen was tender in the right upper quadrant but soft and had no abdominal distension. She had multiple comorbidities which included atrial fibrillation, previous myocardial infarction, left ventricular failure, hypertension, type 2 diabetes mellitus, osteoarthritis, gout, previous urosepsis, bilateral carpal tunnel syndrome, total abdominal hysterectomy, and Achilles tendon lengthening.

**Figure 11:**
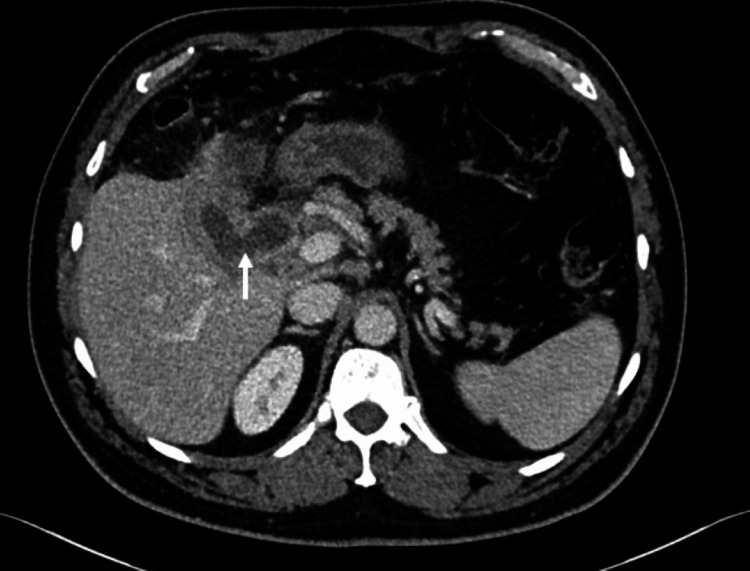
Axial portal venous CT demonstrates a fistula between gallbladder and duodenum (white arrow).

**Figure 12:**
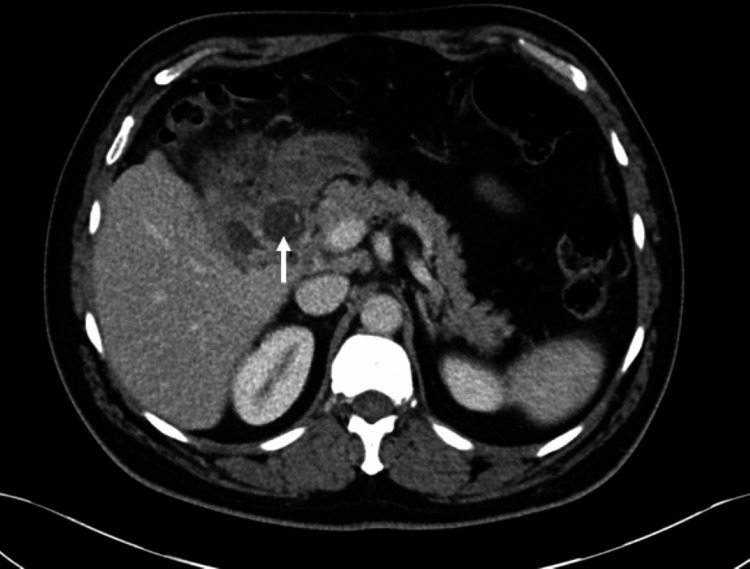
Axial portal venous CT shows a faintly calcified gallstone within the fistula (white arrow).

On admission, her blood test results showed no derangement of U&E and LFT. The other blood test results can be seen in Table 5. She had an ultrasound abdomen on the same day which showed a 26 mm gallstone at the neck of the gallbladder as well as sludge was noted at the base of the gallbladder. There was marked inflammation of the gallbladder wall, demonstrating oedematous changes, with a wall thickness measuring 10.2 mm. Her common bile duct was dilated at 9.8 mm. Her CT scan showed a fistula connecting the gallbladder to the duodenum (Figure 11) with a faintly calcified gallstone in the fistula (Figure 12). She was treated conservatively with antibiotics and was discharged on day 3 of admission with an outpatient laparoscopic cholecystectomy planned.

**Table 5:** Laboratory blood results of patient in case 6. g/L: gram per litre; 10^9^/L: 10^9^ per litre; mg/L: milligram per litre. Hb: haemoglobin, WBC: white blood cell count.

Component	Results	Normal reference range and units
Hb	95	115–165 g/L
WBC count	13.2	4.0–11.0 × 10^9^/L
Neutrophils	10.7	2.0–7.5 × 10^9^/L
C-reactive protein	206	0–10 mg/L

The following month, she was due for her laparoscopic cholecystectomy operation but it was cancelled as the patient had not stopped her warfarin medication prior to surgery and her operation was rescheduled four months later. Since her previous scan showed a dilated common bile duct with some low echogenic foci, a magnetic resonance cholangiopancreatography (MRCP) was requested to rule out the intrahepatic path. MRCP was performed as an outpatient and it showed the appearance of Bouveret’s syndrome with the erosion of the lamellated gallstone from the gallbladder into the proximal part of the duodenum. There was no evidence of common bile duct stones.

Her re-scheduled laparoscopic cholecystectomy was cancelled due to the latest findings of the MRCP result. In order to confirm the diagnosis of Bouveret's syndrome, she had an outpatient OGD a month later, which showed minimal pyloric stenosis with no stones, fistula, or erosion. As the results of the OGD could not fully confirm the suspicion of Bouveret’s syndrome, the patient was treated conservatively and discharged.

## Discussion

Cholelithiasis is a common condition occurring in the general population, 6% in men and 10% in women [5]. While the majority of patients with gallstone-related issues would recover from them without long-lasting complications, a small percentage (approximately 6%) would develop rare complications such as cholecystoduodenal fistula [3]. Fistula formation occurs as a result of adhesions between the gallbladder and bowel wall or stomach due to chronic inflammation, thus causing reduced arterial blood supply and venous drainage. The resulting pressure necrosis and compression from the calculus against the wall of a gallbladder eventually results in the eroding of gallstones through the wall. Gastric outlet obstruction (Bouveret’s syndrome) occurs if impaction is at the pylorus or duodenum, and gallstone ileus if impaction is in the terminal portion of the ileum [2,3].

Bouveret’s Syndrome presents with a clinical picture of gastric outlet obstruction. The patient can present with vomiting, upper abdominal pain, and/or distension of the abdomen. Less commonly, patients present with fever, anorexia, weight loss, signs of dehydration, and hematemesis due to bleeding from the bowel wall and gastroduodenal or celiac artery erosions [2]. Due to the clinical presentations being non-specific and the advanced age of the patients, it is imperative to consider other differential diagnoses that may cause gastric outlet obstruction, including gastric cancer and peptic ulcer disease.

In terms of laboratory results, findings consistent with obstructive jaundice, such as the rises in total and direct bilirubin, gamma-glutamyl transferase (GGT), and ALP are possible, as determined by the anatomical level of obstruction. Leukocytosis and acid-base alterations, including renal failure, can be seen. The severity is determined by the intensity of the inflammatory response, comorbidities, and the individual’s compensatory mechanisms. In gastric outlet obstruction, there is often hypochloraemia and metabolic acidosis with paradoxical aciduria. Elevation of amylase is less commonly reported [2].

The diagnosis of Bouveret’s syndrome is usually confirmed via endoscopy [3]. In 1976, Grove was the first person to recognise the case of gallstone ileus via gastroscopy [6]. In approximately 69% of patients, a stone-causing obstruction can be visualised, sometimes only partly visualised through the duodenal wall, whereas in 31% of these cases, endoscopy showed obstruction without being able to visualise the stone or fistula [3]. In a small proportion of cases, the stones could not be seen because they were only compressing the lumen [3].

Plain abdominal radiography (AXR) might show ectopic gallstone, pneumobilia and bowel obstruction, which is known as Rigler’s triad [2]. In certain scenarios, the migration of a previously known stone may also be detected. The presence of air in the gallbladder may cause air-fluid levels to be observed in the right upper quadrant. If the patients can tolerate oral contrast intake, an oral contrast meal followed by AXR can be beneficial to demonstrate detailed information about the anatomical structures involved, including the site of obstruction [2,3,7].

A CT scan is the best imaging modality to identify the cause of Rigler’s triad. CT is diagnostic in 99% of cases, and it has a sensitivity rate of 93% and a specificity rate of 100% [8]. It often aids in showing the exact level of obstruction, the status of the gallbladder, and the site of the duodenal fistula. However, almost 25% of gallstones cannot be visualised via CT due to their bile/fluid isoattenuation; thus MRCP may be useful to differentiate fluid from calculi and visualises the fistula precisely without the need for oral contrast material [7].

Management involves endoscopic techniques initially and surgery if these are unsuccessful. A myriad of endoscopic techniques have been established, including endoscopic extraction, endoscopic laser lithotripsy (ILL), extracorporeal shockwave lithotripsy (ESWL), and intracorporeal electrohydraulic lithotripsy (IEHL) [3]. These techniques have been reported to be an alternative to surgery for stones causing proximal obstruction, whereas surgery is more commonly performed for gallstones causing ileal impaction [3].

The first line of management for Bouveret’s syndrome is endoscopic treatment. In 1985, the first successful endoscopic extraction was reported by Bedogni et al. [9]. Following this, multiple case reports have been published detailing successful endoscopic management of Bouveret’s syndrome [10-12] with the use of different-sized snares, retrieval nets, baskets, and grasping forceps, among others. It is a highly skill-dependent, time-consuming, and challenging procedure; success rates have been previously established to be less than 10% [13].

The use of endoscopic treatment with a range of different lithotripsy modalities has been described. These techniques may require more than one endoscopic procedural session. There have been several case reports of ILL performed alone and/or in combination with ESWL. Maiss et al. described the first successful use of ILL in 1999; however, it required a total of nine sessions [14]. The disadvantages of this procedure include the need for multiple sessions and an increased risk of converting a proximal gallstone obstruction into a distal gallstone obstruction due to partial stone fragmentation [3]. IEHL can be done on its own or in combination with other methods. In 1991, Moriai et al. removed two 3 cm gallstones using IEHL and mechanical lithotripsy. In 1997, Dumonceau et al. successfully treated Bouveret’s syndrome with IEHL after the failure of ESWL [15,16]. The drawback of this method lies in its mechanism of action; unintentionally focusing shockwaves onto the intestinal wall may cause perforation, peritonitis, and bleeding [3]. ESWL has been used to successfully treat patients with Bouveret’s syndrome. However, it has its own limitations, such as the need for several return sessions and eventually requiring the use of endoscopy. It may also be challenging to perform this procedure in obese patients or if there are gas-containing bowel loops seen in between the gallbladder and abdominal wall [17-18]. Endoscopic extraction is often used depending on the size of the gallstones. If stones are larger than 2.5 cm, it may pose a challenge to extract them endoscopically, despite literature reporting extractions of stones up to 3 cm [13]. These stones have a hard outer shell and a soft inner core, which makes mechanical fragmentation more difficult via the use of endoscopic lasers or forceps. Instead, larger stones can cause ischaemic ulceration of the duodenal wall.

Failing endoscopic therapeutic procedures, surgical management is the only option for these patients. Approximately 42% of these patients have been previously noted to have undergone failed endoscopic treatment prior to having the surgery [3]. Surgical options that are available are a combination of enterolithotomy (removal of the stone) with cholecystectomy and fistula repair [3]. This can be performed via open surgery, totally laparoscopic or laparoscopically assisted procedures.

During the surgical operation, the entire intestine should be examined as 16% of cases revealed the presence of other gallstones present at another site in the digestive tract [19]. It is preferred that the stone should be removed in the stomach through a gastrostomy, or if this is not possible, removal of the stone in the small bowel can be done via an enterotomy [20-22].

Fistula repair in patients managed with endoscopic methods or enterolithotomy is often unnecessary as fistulas often spontaneously close, especially when the patients’ cystic duct is patent and no residual stones remain [3]. A number of fistulas spontaneously close within 30-60 days, while some still remain active 90 days post-initial treatment [2]. Nevertheless, factors such as persistence of symptoms, the likelihood of recurrence, and the risk of gallbladder cancer would justify the need for fistula repair in specific cases [23].

## Conclusions

Bouveret’s syndrome is a rare and challenging cause of gastric outlet obstruction and gallstone ileus. This diagnosis should be included in the differentials in elderly patients with no other possible cause presenting with small bowel obstruction. If deemed appropriate, patients should have definitive treatment immediately to avoid further complications. Endoscopic approaches are the preferable first line of management and have an increasingly higher success rate in the modern era. The use of mechanical and intracorporeal lithotripsy permits the removal of larger stones and should be considered the first line of a therapeutic plan for this rare condition.
